# Evolution, perception, and the mind

**DOI:** 10.1007/s10339-024-01208-x

**Published:** 2024-08-19

**Authors:** Jerome A. Feldman

**Affiliations:** University of California, Berkeley, USA

## Abstract

The classical mind–body problem persists as one of the deepest scientific mysteries. Despite the grand claims of the new AI, some of the most basic facts of human vision cannot be explained by current or proposed theories of brain structure and function. This paper reviews some well-known mysteries including the neural binding problem, blind sight, subjective experience and prosthetics. There is continuing progress, but the core mysteries of the mind seem to require fundamental advances for any reductionist resolution.

## Text

Recent advances in several fields have led to increased concern with what science can learn about the *mind*. The classical mind–body problem persists as one of the deepest scientific mysteries. A search of Google Scholar for “mind–body” yields about a million links to articles.

This paper will present some evidence of why current science is inadequate to explain the relation between subjective experience and bodily activity. We will briefly review some promising research that helps unravel the mind–body problem but does not resolve it. And finally, we will suggest that it is likely that fundamentally different approaches will be necessary to make notable progress in solving the mind–body problem. In other words, a scientific revolution will be needed.

In addition to the many profound aspects of the mind, such as Consciousness, emotion, qualia, self-awareness, dreams, and free will, there are also profound *perceptual* mysteries that arise whenever we open our eyes. These are simple examples of the mind–body problem. You can experience this for yourself. Close or cover one eye,
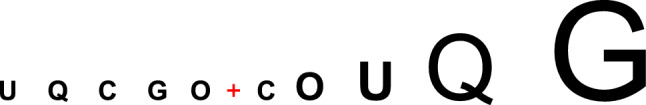


then focus on the + , and try to read the letters to the left. You will find it much easier to read the letters on the right. Neural encoding of fine detail is restricted to the fovea, covering about the size of your thumbnail at arms-length. Our mental experience of a stable high-resolution scene is i*nconsistent* with conventional science of the visual system, as well as any proposed alternative (Feldman 2012). This is a simple example of the mind–body problem and confounds any reductionist theory of mind.

The currently popular attempts to reduce 1st person cognition to neurons, such as (Sapolsky 2023) or physics (Carroll 2017) totally ignore the mind and fail to demystify even our most basic experience, including perception as above, These works are all based on the unsupported assumption that subjective (1st person) mentation needs no scientific explanation.

## Evolution

Nothing in Biology Makes Sense Except in the Light of Evolution (Dobzhansky 2013).

This paper explores some mysteries of perception in the light of Evolution. It turns out that evolution has a great deal to say about why perception is not a veridical mapping of the world, but does not explain the mechanism of subjective (1st person) experience as seen in the initial demonstration above.

For most of its history, Cognitive Science developed without the benefit of the science of evolution. This is changing, largely due to Eco-E*vo-Devo* https://en.wikipedia.org/wiki/Ecological_evolutionary_developmental_biology, which inherently links the evolutionary fitness of an animal (including autonomous cells) to its internal structure and function (Gilbert 2015; Carroll 2017). For convenience, I will use the shorthand “evolution + ” for Eco-E*vo-Devo Evolution*. In addition, the science of any animal phenomenon must include its evolutional + history.

There is a convincing and expanding evolutionary + story on the emergence of complex molecules and life (Mitchell 2023), although with some gaps. Since we are ultimately interested in the human mind, we start with living animals, including autonomous cells. The discussion will follow an evolutionary + approach (Cisek 2021) to the subjective experience of animals.

The classical mind–body problem remains mysterious for a basic reason. There is an unescapable dichotomy – the mind (subjective experience, 1st person) is private. There is no privileged access to the mind of another animal. In general, *Perception*, is the mechanism by which the animal body, and presumably its mind (if any) acquires useful information about the external world. Any subsequent response depends entirely on the animal’s internal information and processes.

Over the years, scientists have developed powerful conceptual tools for exploring the relationships between the structure and function of an animal and its physical and social environment. *Umwelt* (Jacobs 2004; Von Uexküll 2001) is defined as those aspects of the environment that are accessible to the animal. *Affordances* (Gibson 1977) are the aspects of the Umwelt that are potentially usable by the animal. *Autopoesis *(Maturana 1991*)* describes the necessity to study the animal in terms of its internal structure and function, which is all that the animal can use. This is extended to theories of *structural coupling* with the Umwelt. From this perspective, the role of *perception* is to supply an animal with useful information about the external and internal milieu. Of course, the effective internal mechanisms include emotions, hormones, microbes, etc. (Yong 2016). Subjective experience (mind) arises from the results of perception and is not a veridical rendition of the world.

*Evolution* + (in the extended sense of eco-evo-devo (Gilbert 2015) inherently links the fitness of a living creature in context to its internal structure and function. It seems appropriate to start with animals and their prototypical protozoan ancestors, the Amoeba. There is a wide range of amoebic forms including our white blood cells. Free living “Amoeba Proteus” are the most commonly studied amoeba and are prototypical single cell animals. They are characterized by (Damasio 2010).

“All living organisms from the humble amoeba to the human are born with devices designed to solve automatically, no proper reasoning required, the basic problems of life. Those problems are finding sources of energy; incorporating and transforming energy; maintaining a chemical balance of the interior compatible with the life process; maintaining the organism’s structure by repairing its wear and tear and fending off external agents of disease and physical injury. “

*Perception* is the mechanism through which an animal gains information to address these needs. Most of the research on perception, including this paper, focuses on vision, but there is a wide range of other modalities. An exceptional overview of animal perception is the prize-winning book by Ed Yong (2023). Among the modalities discussed are smell, taste, color, pain, heat, contact, flow, vibrations, sound, echoes, electric fields, and magnetic fields.

The earliest animal perception is chemical, as an extension of internal, chemical processes (Jacobs 2021). The two original senses are smell and taste, in water and in air. Importantly, early *perception* evolves as the animal interacting with specific molecules in ways that promote fitness. The triggering molecules from the *Umwelt* are distinct from internal structures. So, from the outset, perception does not directly model the external world, but evolves internal responses to *affordances*. This is the evolutionary + foundation for the difference between world features and their representation in animal brains. *Autopoesis* shows that any decisions and actions resulting from perception, including interoception, must be calculated internally.

One informative example is the color constancy of the Hawkmoth. These moths recognize the same flower color under changing illumination conditions. This ability is especially important for foragers that act under a wide range of lighting conditions. Receptor adaptation contributes a large part to color constancy; the sensitivity of specific receptors decreases as a result of adaptation of the photoreceptor cells being stimulated by the background illumination. Importantly, color constancy evolved by natural selection; moths with better vision tend to prosper. No learning by the moth is needed. This is a simple example of evolution inducing an evolutionary + adaptive internal structure. For people, dealing with our much more adaptive and complex Umwelt has been an important factor in the evolution + of the mind.

Perception also has active mechanisms, like human saccade and Curollary Discharge (Crapse and Sommer 2008). There is the wide spread mechanism of corollary discharge (TB Crapse, MA Sommer—Nature Reviews Neuroscience, 2008—nature.com) where the perception mechanism includes information on the agent’s own actions, including emission of sonar (bats, dolphins) and reflections of human breathing in some blind people.

The analysis above provides a grounding for the origin of the mind–body problem. The (evolutionary +) fitness of a simple animal (e, g. a cell) depends on its ability to perceive and act on Umwelt affordances. There is a positive feedback loop. Evolving + a new perception involves also new internal change, which can lead to enhanced functionality. The internal functionality is not a veridical capture of the source of sensation, but something (~ 1st person) that improves fitness.

A cell has no unifying sense of itself (~ mind). The minimal requirement for any kind of self-awareness, seems to be a brain-stem (Merker 2007). An extended presentation on the evolution + of subjective experience, including a literature review can be found in (Feldman 2020).

### Current efforts

#### The binding problem and the new AI

**Fig. 1:**
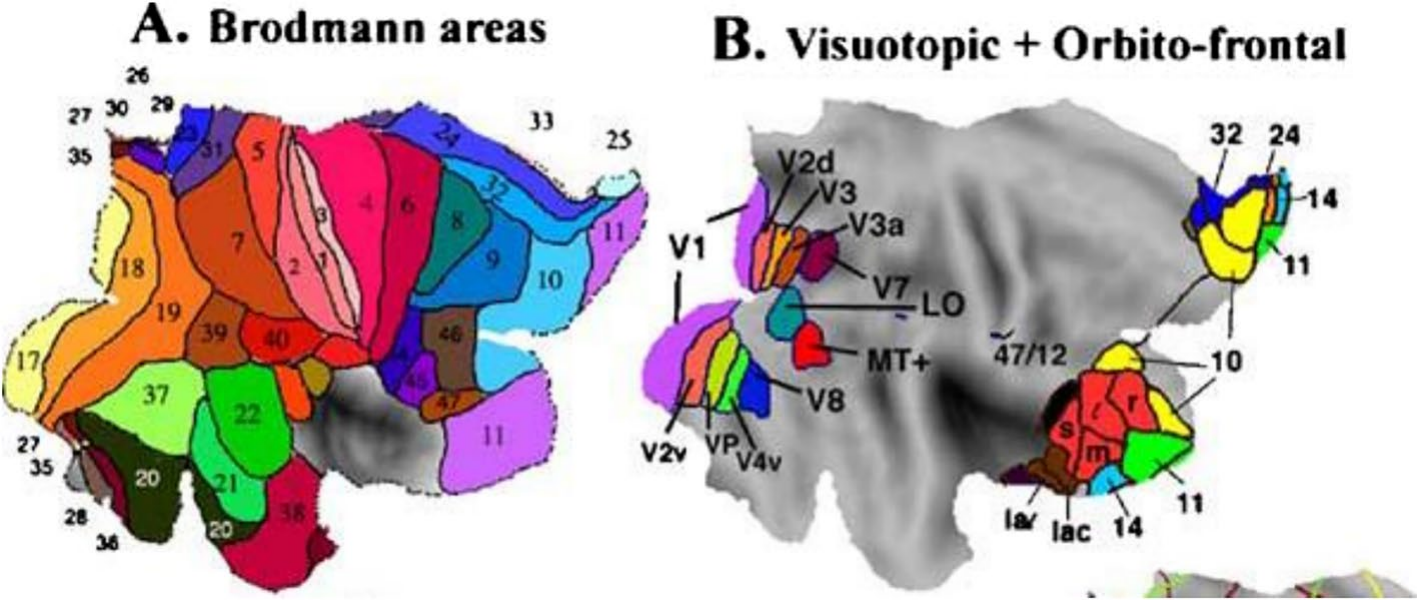
Flat map projection of the Human brain (5)

We return to the initial demonstration mystery of everyday vision. You may have read that vision works because our eyes shift (saccade) about three times a second. This is remarkably rapid, but is much too slow and sparse to explain our experience whenever we open our eyes. There are two basic mysteries—how brains encode the perceived detailed scene and how we become subjectively aware of this. Perceptual mysteries are particularly striking because science has extensive knowledge of visual psychophysics and of the primate visual system. For example, Fig. 1.

Figure 1A is a standard flattened projection of one hemisphere of the human brain with the various areas colored. The numbers refer to the traditional Brodmann classification of brain regions from their anatomical details. Modern methods have further refined this picture and elaborated the basic functions computed in these different areas. The visual area V1, our main concern for the stable world experience, is shown as the yellow area on the left of Fig. 1A (as area 17) and as the magenta area in Fig. 1B. You can see from Fig. 1A that the primary function of the various cortical areas is well known – there is no space for the large detailed map that we subjectively experience. Figure 1B provides more detail on this functional separation in the visual system, which is at the core of the neural binding problem, another perceptual mystery. An anytime reminder of the mind–body problem is to view your surrounds and try to explain the substrate of your experience in terms of Fig. 1.

More generally, focusing on perception yields a major advantage in mind–body studies. Many mysterious findings occur in vision, where the stimuli, the neural substrate, and the subjective experience are scientifically accessible. As we will discuss below, there are already important scientific results, and even some clinical applications (Fox 2020), in human perception and prostheses.

Another ancient mystery of perception is called the “neural binding problem”, NBP (Feldman 2013). There are several versions of the problem, but the most basic one can be seen from Fig. 1. Focusing on Fig. 1B, we can see that there are multiple specialized visual areas in the back of the brain. For example, the area V4 encodes color and the area MT encodes basic motion information. The most basic NBP is how we simultaneously experience a static red circle and a moving blue square, without interference. A simple version of this NBP can be explained by pairing the time and place of occurrence of visual features, but the general problem of the unified subjective experience (above) remains unsolved.

A more complex NBP arises in language. All visual animals need feature binding, but variable binding mainly arises in language and other symbolic thought. As a simple case, consider the sentence “He gave it to her before”. All of the six words are variables and need to be bound to meaning choices for the sentence to be understood. The core problem is that the meaning of each word can depend on the context before or after (e.g., German) the sentence. Meaning can also depend on general knowledge and the current situation. There is still no satisfactory solution to the symbolic NBP. More generally, the context problem in language is studied as “long distance dependency”.

https://www.sciencedirect.com/topics/computer-science/long-distance-dependency. This has no known classical solution.

Of course, the main stream of work on language processing is currently focused on machine learning and connectionist neural models. From the outset in the 1980s, Neural models have been limited to explicit connections between units. In addition to major improvements in computational speed and storage, considerable progress has resulted from advances to computational *structure*. Until recently, connectionist models (now called AI) suffered from the same basic NBP, long-distance dependency, above.

Machine learning and AI researchers have been aware of the NBP problem and have investigated various architectures to overcome it (Greff 2012), The major break-through, *transformers and attention*, occurred in 2017 and continues to drive progress. Transformers and attention comprise a method for uniting representations for distant words, even for distinct computations like input and output for machine translation. It depends on earlier AI advances and massive amounts of data and processing. Essentially, Transformers learn representations for every combination in a huge corpus, constrained by locality and similar statistics (attention). Current systems can generate impressive looking responses to input, but also make many errors. In fact, attention alone does not suffice and applications involve many other functions including human input to the pre-trained systems (Ouyang 2002). Moreover, the basic problem of context dependence remains unsolved at scale (Ding 2024).

Variants of transformers continue to have increasing impact beyond language. Increasingly, the term AI is less appropriate than “machine learning”. The general idea of training systems on examples has been extended to recursively training aspects of the system and even the methodology. Over a wide range of domains, this technology is able to learn significant features and group instances with similar patterns. This can lead to finding novel combinations (e, g., drugs) that are similar to known useful ones.

This success has even rekindled the concern for super-human general intelligence. The rapidly expanding field of Machine Learning, also called Artificial Intelligence (AI) is currently seen as epochal. An excellent book on the impact of new AI on many aspects of society is (Castells 2024).

## Demystification

This article focuses on scientific aspects of *subjective* e*xperience*. One important step is to identify phenomena that are currently viewed as *mysterious*. The goal is to identify phenomena and methodologies that support *demystification.* Although this goal is not commonly articulated, there are several existing demystification advances which are yielding important scientific and clinical results.

One current mental demystification involves blindsight (Fox 2020), until recently considered a major mystery. People with certain deficits are unable to report what they see, but can carry out appropriate actions like grasping a tool. In retrospect, the mystery of blindsight arose from the simplistic assumption that visual perception was a single integrated function. Actually, there are several interconnected vision networks, including some, like reflexes, that do not require conscious perception. The exploration of multiple visual systems is also having clinical applications (Fox 2020). This work has been extended to suggest different therapies for patients with separate histories.

In a few remarkable cases (Iriki 2012), we also know the neural correlates of the embodied experience of external objects like the blind man’s direct sensing of the cane tip. For many years, tool embodiment was treated as a deep mystery. In a 1996 experiment, Iriki et al. trained macaque monkeys to retrieve distant food using a rake, and recorded neuronal activity in the caudal postcentral gyrus where the somatosensory and visual signals converge (Iriki et al. 1996). There they found a large number of bimodal neurons, which appeared to code the body schema of the hand. After extensive tool use, the visual receptive fields of these cells adapted to include the entire length of the rake or cover the expanded accessible space. This provides a direct example of induced subjective experience.

Subsequent developments have included attempts to improve human prostheses by generating this kind of subjective embodiment (D’Anna 2019, Shu et al. 2024). With training, people can improve performance by developing direct embodiment of a prosthetic arm with indirect feedback or, better, with direct connection to appropriate nerves. Of course, the *mechanism* of subjective experience is still unknown.

Taken together, these disparate studies of subjective experience comprise a starting point for an ecologically sensitive science of subjective experience of perception and the mind–body problem. An ambitious outline of such a science is recent work by (Iriki 2012), as an extension of his seminal work (Iriki et al. 1996) on the neural basis of subjective experience.

## Conclusions

“*The most beautiful thing we can experience is the mysterious. It is the source of all art and science*.” *Einstein*

The evolutionary + approach makes clear why subjective experience is not based on a veridical model of the external world or the internal milieu. An animal has no privileged access to the physical and social world or to its own detailed operation. However, the animal’s perception and actions evolved + to support fitness. The mind evolved + because an animal needs a strictly internal mechanism to relate perceptions (including interoception) to action. For animals with subjective experience, like us, the mind (whatever that is) is part of the process.

Consider again the initial example of how we experience vision. It is amazingly more adaptive than the known neural substrate could support. There are two deep mysteries: how the body computes the subjective model and also how we become aware of it. We lack not only a theory of what is real, but also any coherent understanding of 1st and 3rd person experience.

The many remaining mysteries of the mind strongly suggest that any reductionist solution of the mind–body problem will require scientific reconceptualization on the scale of general relativity or quantum theory. Both of these groundbreaking theories are famously unintuitive and any physical explanation of the mind, if one is discovered, may well be equally challenging.

For now, a concrete description of the mysteries of perception can be expressed by a Gödel-like *finding* “No existing or proposed neural theory of the brain is *consistent* with the everyday experience of vision.” This is not a theorem and is subject to refutation at any time. Its current validity is an instance of the mind–body problem. However, as discussed above, taking the *finding* as a working hypothesis does provide an ideal scientific domain at the boundary of the tractable and the mysterious.
